# Differential target of oxidative damage in *Helicobacter pylori-*related and autoimmune atrophic gastritis: a single-center study

**DOI:** 10.3389/fmed.2025.1583833

**Published:** 2025-06-04

**Authors:** Filippo Pelizzaro, Romilda Cardin, Gemma Maddalo, Michela Palo, Milena Minotto, Chiara Carlotto, Matteo Fassan, Fabio Farinati, Fabiana Zingone

**Affiliations:** ^1^Gastroenterology Unit, Camposampiero Hospital, ULSS 6, Camposampiero, Italy; ^2^Department of Surgery, Oncology and Gastroenterology, University of Padova, Padova, Italy; ^3^Gastroenterology Unit, AULSS 2 Marca Trevigiana, Treviso, Italy; ^4^Department of Medicine, University of Padova, Padova, Italy; ^5^Veneto Institute of Oncology, Padova, Italy; ^6^Gastroenterology Unit, Azienda Ospedale-Università Padova, Padova, Italy

**Keywords:** autoimmune atrophic gastritis, multifocal atrophic gastritis, gastric cancer, oxidative stress, genomic damage, 8-hydroxydeoxyguanosine

## Abstract

**Introduction:**

Oxidative stress, which characterizes inflammatory and autoimmune diseases, is involved in atrophic gastritis progression. We aimed to compare the presence and patterns of oxidative stress in autoimmune atrophic gastritis (AAG) and multifocal atrophic gastritis (MAG), investigating its role in disease progression and the development of genomic damage.

**Materials and methods:**

In this study, 120 consecutive patients with atrophic gastritis (70 AAG and 50 MAG) were collected. Serum/plasma dynamic reactive oxygen metabolites (dROM test-spectrophotometry), nitric oxide (NO-ELISA), advanced oxidation protein products (AOPPs—spectrophotometry), tissue levels of cytokines (IL-10 and TNFα–ELISA), and 8-hydroxydeoxyguanosine (8-OHdG) (HPLC-ED) were evaluated.

**Results:**

No significant differences in dROMs, NO, and AOPP levels were demonstrated between AAG and MAG. In AAG patients, those with early atrophy exhibited higher levels of dROMs compared to those with advanced stages (*p* = 0.03), and those with early ECL-hyperplasia (ECL-H) exhibited higher dROMs and AOPP levels compared to those with advanced hyperplasia (*p* = 0.02). 8-OHdG levels were significantly higher in MAG patients than in AAG patients (*p* = 0.001). A ROC curve showed that patients with low 8-OHdG levels had a very low OR of belonging to the MAG group. A positive linear correlation was observed among serological biomarkers of atrophy and dROMs (*p* = 0.004). IL-10 was higher in patients with MAG vs. those with AAG (*p* = 0.008), and it was also higher when patients were sub-grouped according to the OLGA stages (*p* = 0.01).

**Conclusion:**

Inflammatory responses and oxidative stress characterize atrophic gastritis, irrespective of etiology. However, a boost in inflammation-induced genomic damage is observed only in MAG, since AAG showed significantly lower 8-OHdG levels. Therefore, oxidative stress seem to play a minor role in the development of carcinoid and gastric cancer in AAG, and this finding may explain the lower risk of tumors in these patients.

## Introduction

Autoimmune atrophic gastritis (AAG) is a chronic immuno-mediated inflammatory gastric disease, leading to a progressive destruction of the gastric body and fundus glands (1, 2). AAG is characterized by the recruitment of sensitized T cells and results from a complex interaction between autoantibodies directed against H+/K+ ATPases (anti-parietal cells autoantibodies, APCA) and intrinsic factor (anti-intrinsic factor (AIF) antibodies) (1). From a pathophysiologic point of view, the main feature of AAG is the progressive loss of acid output, which results in mucosal damage (2). Multifocal atrophic gastritis (MAG) is a distinct form of gastric damage, where *Helicobacter pylori* (*H. pylori*) infection has been recognized as the primary etiologic agent that initially causes an antral-predominant damage and then a “pangastritis”, involving the whole stomach due to a long-standing infection (3). Both forms of gastritis are characterized by atrophic changes and metaplastic rearrangement, with an increased risk of neoplastic complications (4–7), which is higher in MAG compared to AAG.

The OLGA staging system is an international classification of gastric atrophy, combining the presence of antral and body atrophy in an analogic score (8, 9), that predicts the risk of neoplastic progression to dysplasia (i.e., intraepithelial neoplasia) or gastric adenocarcinoma (GC), according to Correa's cascade of events (10). In addition, AAG presents a substantial incidence of type I gastric carcinoid, a neuroendocrine tumor with a benign behavior in the majority of cases, which develops as a consequence of achylia, hypergastrinemia, and progressive enterochromaffin-like cell hyperplasia (ECL-H) (4–6).

In these processes, oxidative stress appears to play a central role. Oxidative stress occurs due to an imbalance in the production and removal of free radicals, i.e., hyper-reactive atoms or molecules with an external unpaired electron, including reactive oxygen and nitrogen species (11). In recent times, oxidative stress has been considered a causative element of several diseases due to its role in maintaining chronic inflammation, and it is also involved in promoting autoimmune diseases and in causing cancer, as a consequence of protein and genomic damage due to the accumulation of free radicals (12). For instance, the advanced oxidation protein products (AOPPs), first described by Witko-Sarsat et al., are primarily derived from the reaction of plasma proteins with oxidative compounds, their accumulation depending on an imbalance in formation, repair, and clearance of the protein substrate (13, 14). AOPPs are described in the literature as substances implicated in autoimmunity, since they induce the formation of oxidatively modified antigens, with subsequent exposure of these neo-antigens and, in turn, autoantibodies generation (15–18). AOPPs have also been described in cancer, and, for the stomach, their levels are reportedly higher in GC compared to controls (19). Eight-hydroxydeoxyguanosine (8-OHdG) formation is the most relevant type of oxidative genomic damage implicated in carcinogenesis (12). Our group described the progressive accumulation of 8-OHdG in chronic atrophic gastritis compared with controls, in a pivotal study (20, 21).

The rationale of this study was represented by the hypothesis that, as with any other type of inflammatory disease, gastritis can be characterized by increased tissue-free radical production. Several studies demonstrated an association between oxidative stress caused by *H. pylori* infection and the presence of macrophagic infiltrate in the gastric mucosa. Macrophages are largely involved in oxidative stress by generating free radicals through the “respiratory burst” (22). To the best of our knowledge, there are no studies on oxidative stress and related damage in the development of AAG and its progression to cancer. This study aims to assess the extent of oxidative stress in both AAG and MAG and to identify any difference in the targets of this oxidative stress, in relation to the different risks of progression to adenocarcinoma in the two types of gastritis.

## Patients and methods

In this prospective study, 70 AAG and 50 MAG patients, managed at the Gastroenterology Unit of the University of Padova Hospital, between 2017 and 2018 were included. The study was performed in accordance with the Declaration of Helsinki, with the approval of the ethics committee at our institution (CESC approval 3312/AO/14 prot. 0034435), and all patients subscribed to an informed consent before being enrolled in this study. Patients with a history of smoking, alcohol abuse, cancer, and other diseases were excluded.

The biopsy sampling during upper gastrointestinal endoscopy followed the Sydney System with two biopsies from the antrum, 1 from incisura angularis, and 2 from the body. In addition, biopsy was also performed for each visible lesion. For the specific evaluations of this study, two more biopsies were performed from the antrum in MAG and the body in AAG and stored at −80°C until analysis. For each patient, a total of 10 mL of peripheral blood was collected and 4 mL of EDTA or 8.5 mL of integrated separator was added. The samples were then centrifuged and stored at −20°C until they were used.

Laboratory data regarding gastric function were collected for all patients. Specifically, pepsinogen I (PGI), pepsinogen II (PGII), total gastrin, chromogranin A, and the presence of APCA and AIF were registered in AAG patients.

We classified patients as *H. pylori* positive when *H. pylori* antibodies were positive, in case of positive histology, or when previous infection was well documented in their clinical history, in both MAG and AAG patients. Given that most recruited patients were in an organized follow-up, only one patient with active *H. pylori* infection was identified among those with autoimmune atrophic gastritis, while six patients with active *H. pylori* infection were identified in the group of MAG patients.

### Reactive oxygen metabolites (dROMs)

To determine dROMs, which refers to derivatives of free radicals, we used spectrophotometry at 540 nm, using the dROM test (Diacron International, Grosseto, Italy) according to the manufacturer.

The test results were expressed in U CARR, obtained by applying the formula:


$$\begin{array}{l} \text{U CARR }=\text{ Abs sample}/\text{Abs calibrator }\times \;\left[\text{calibrator}\right]. \end{array}$$

Abs sample and Abs calibrator are the absorbance values measured in the sample and calibrator, respectively, and [calibrator] is the concentration of the lyophilized calibrator used, which had a reference value of 300 U CARR (1 U CARR = 0.08 mg H_2_O_2_/dL).

Values expressed as <320 UCarr, 321–340 UCarr, 341–400 UCarr, and >400 UCarr were considered borderline, mild, moderate, and high, respectively.

### Advanced oxidation protein products (AOPPs)

Plasma AOPP levels, expressed as μmol/L of chloramines-T equivalents, were measured by the spectrophotometric method performed by Witko-Sarsat (13).

### Nitric oxide (NO)

Serum NO levels were determined spectrophotometrically by measuring the accumulation of its stable degradation products, nitrate and nitrite, using a commercially available assay (Oxford Biomedical Research, USA). NO levels were expressed in μmol/L. Based on the literature data, NO levels of <0.1mM were considered normal (23).

### Cytokine expression in tissue

For protein extraction, a lysis buffer supplemented with a cocktail of protease inhibitors (Sigma-Aldrich, Milan, Italy) was added to the samples. Subsequently, the samples were homogenized, and supernatants were stored at −80°C until use. Protein concentration in biopsies was quantified by BCA Protein Assay (Thermo Scientific Pierce, Rockford, Illinois, USA) with spectrophotometric absorbance samples at 540 nm. For the determination of interleukin 10 (IL-10) and TNFα in the gastric mucosa, the samples were prepared according to the instructions provided by the manufacturer (Human IL-10 ELISA kit and Human TNFα ELISA Kit—Immunological Sciences, Rome, Italy). The absorbance was then read by spectrophotometry at 450 nm. The diluted concentration of IL-10 and TNFα was multiplied by the dilution factor. The IL-10 and TNFα values are expressed in pg/mL.

### 8-hydroxydeoxyguanosine (8-OHdG)

This assay was carried out as described in our previous studies in three steps: (1) genomic DNA extraction using Wizard Genomic DNA Purification Kit (Promega Italia, Milano); (2) nuclease P1 and alkaline phosphatase hydrolysis of DNA; and (3) 8-OHdG determination using an HPLC equipped with an electrochemical detector (HPLC-ED) (ESA Coulochem II 5200 A, Bedford, MA). The 8-OHdG levels were expressed as the number (no.) of 8-OHdG adducts/10^5^ deoxyguanosine (dG) (20).

### Pathological evaluations

Paraffin-embedded biopsies were evaluated by expert pathologists, and the atrophy stage was classified according to the OLGA staging system (8). The presence of *H. pylori* infection was evaluated using H&E, Alcian-PAS, and Giemsa for *H. pylori*.

Based on the risk of developing gastric cancer in MAG and of developing also neuroendocrine tumor in AAG, patients were subclassified in “early” OLGA stages (MAG OLGA 0–2 and AAG OLGA 0/1, respectively) and in “advanced” OLGA stages (MAG 3/4 and AAG OLGA 2–4). In addition, AAG patients were further sub-stratified based on the presence of neuroendocrine alteration defined as H-ECL, according to the following classification: 0=absent, 1 = linear, 2 = micronodular, 3 = nodular, 4 = adenomatoid, and 5= carcinoid (24). Patients with “early” H-ECL presented at stages 0–2, whereas those with “advanced” H-ECL presented at stages 3–5.

### Statistical analysis

In the comparison of quantitative data, unpaired *t*-test, Kruskal–Wallis, and Mann–U-tests were used as appropriate. Simple regression and Spearman's rank correlation were used for correlations between variables. The ROC curves were used to identify a cut-off of 8-OHdG values for differentiating between AAG and MAG patients. For all the analyses, SPSS (Statgraphics) was used, and *p-*values of < 0.05 (two-tailed) were considered significant.

## Results

### Epidemiology and clinical features

The baseline characteristics of patients included in the study are shown in Table 1. Compared to MAG patients, AAG patients were younger (mean age 60.9 vs. 69.7, *p* = 0.0007) and more predominantly female (83% vs. 54%; *p* = 0.0006). As expected, past or present *H. pylori* infection was significantly higher in patients with MAG (85% vs. 38.6%; *p* < 0.0001). In 15% of MAG patients, although MAG can be considered a consequence of previous exposure to *H. pylori* by definition, there was no active *H. pylori* infection or a clear clinical history of *H. pylori* eradication.

**Table 1:** Baseline clinical and pathologic features.

**Patients' clinical and pathologic features**	**AAG (*n =* 70)**	**MAG (*n =* 50)**	***p***
Age (mean ± SD)	60.9 ± 11.5	69.7 ± 10.5	0.0007
*H. pylori* positive – n (%)	27 (38.6)	42 (85.0)	<0.0001
Female sex	58 (83.0)	27 (54.0)	0.0006
PCA	63 (90.0)	–	–
IFA	40 (57.0)	–	–
**OLGA staging**			
0–1	11 (15.7)	16 (32.0)	0.03
2	50 (71.4)	23 (46.0)	0.0001
3	9 (12.9)	11 (22.0)	0.22
**H-ECL**			
0	12 (172.)	–	–
1–2	17 (24.3)	–	–
3–5	41 (58.5)	–	–

AAG, autoimmune atrophic gastritis; MAG, multifocal atrophic gastritis; SD, standard deviation; PCA, anti-parietal cell antibodies; IFA, anti-intrinsic factor antibodies; OLGA, Operative link on gastritis assessment; H-ECL, enterochromaffin-like cell hyperplasia.

The majority of patients in both groups had an OLGA stage II, but the proportion of patients in this stage was significantly higher in AAG patients compared to MAG patients (71.4% vs. 46%; *p* = 0.0001). No patients in either group presented an OLGA stage IV gastritis. While no dysplastic or neoplastic lesions were observed among AAG patients, dysplasia at histologic examination was observed among 11 patients (22%) with MAG.

### Markers of oxidative stress in AAG and MAG

No significant differences in dROMs, NO, and AOPPs between AAG and MAG patients were demonstrated, although there was a trend toward higher values of AOPPs in AAG (*p* = 0.09) (Table 2).

**Table 2:** Levels of oxidative stress in AAG and MAG patients.

**Variables**	**AAG**	**MAG**	***p***
dROMs (U) – mean ± SD	336.6 (± 76.8)	312.5 (± 73.2)	0.20
AOPPs (μM) – mean ± SD	72.9 (± 19.3)	63.5 (± 24.1)	0.09
NO (μM) – median (IQR)	179.5 (65.2)	178.7 (44.9)	0.81

AAG, autoimmune atrophic gastritis; MAG, multifocal atrophic gastritis; AOPPs, advanced oxidation protein products; NO, nitric oxide.

Nevertheless, in AAG patients, dROMs levels were significantly higher in the “early” OLGA stages compared to the “advanced” OLGA stages (*p* = 0.03), as well as when compared to the “early” MAG stages (*p* = 0.03). In addition, in AAG patients, dROMs levels were significantly higher in the “early” stages of ECL-H than in the “advanced” stages of ECL-H (*p* = 0.02) (Table 3). Similarly, AOPPs levels were significantly higher in AAG patients without ECL-H compared to those with any stage of ECL-H (*p* = 0.008) and in patients with low ECL-H stages (0–2) compared to patients with high ECL-H stages (3–5) (*p* = 0.02). No difference was detected in the levels of the markers between the groups of patients with and without dysplasia.

**Table 3:** Differences in mean levels of dROMs in AAG and MAG patients at different stages of disease.

**dROMs (U)**	**Mean**	**(±S.D.)**	***p*-value**
AAG early OLGA (0–1)	381.9	(71.3)	0.03
AAG advanced OLGA (2–4)	327.9	(60.1)	
AAG early OLGA (0–1)	381.9	(62.4)	0.03
MAG early OLGA (0–2)	317.6	(47.6)	
H-ECL 0–2	351.7	(78.8)	0.02
H-ECL 3–5	300.2	(57.9)	
AAG *H. pylori* positive	339.8	(81.5)	n.s
AAG *H. pylori* negative	365.8	(62.8)	

AAG, autoimmune atrophic gastritis; MAG, multifocal atrophic gastritis; OLGA, Operative link on gastritis assessment; H-ECL, enterochromaffin-like cell hyperplasia; CI, confidence interval.

### Genomic damage: 8-OHdG

**Figure 1:**
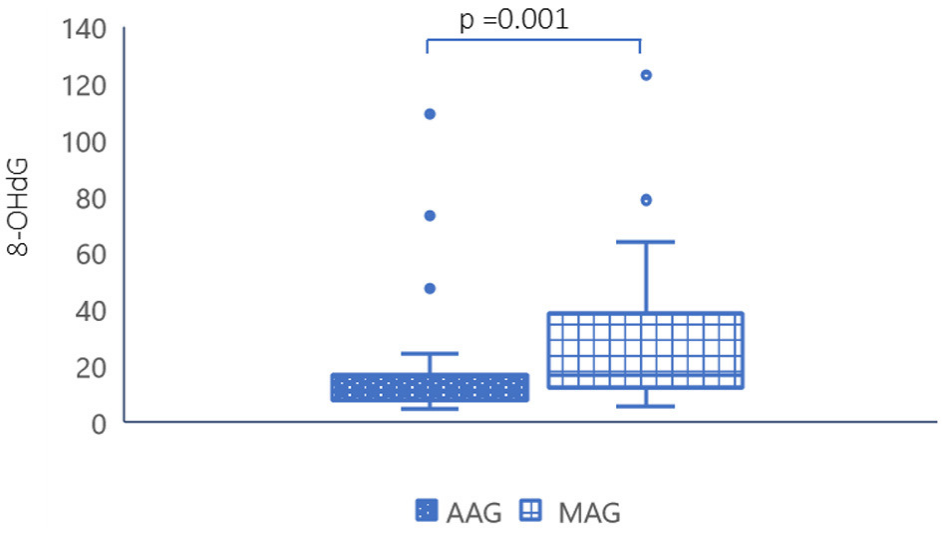
Levels of genomic damage in AAG and MAG patients. AAG, autoimmune atrophic gastritis; MAG, multifocal atrophic gastritis; 8-OHdG, 8-hydroxydeoxyguanosine.

The adduct 8-OHdG, evaluated in tissue samples, was statistically significantly lower in AAG patients (median 10.75 no. adducts/10^5^dG, CI 95% 4.6–24.7) compared to MAG patients (median 16.2 no. adducts/10^5^ dG, CI 95% 5.7–63.1; *p* = 0.001) (Figure 1).

**Figure 2:**
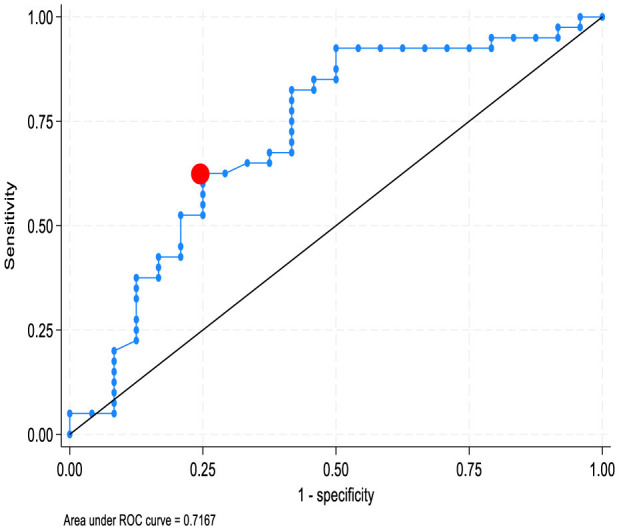
Area under ROC curve to identify a cut-off value of 28.1 (no. adducts/10^5^dG) for 8-OHdG values in differentiating between AAG and MAG patients. AAG, autoimmune atrophic gastritis; MAG, multifocal atrophic gastritis; 8-OHdG, 8-hydroxydeoxyguanosine.

A cut-off of 28.1 for 8-OHdG was observed to distinguish between AAG patients and MAG patients, yielding an AUC of 0.72, sensitivity of 50%, specificity of 92.5%, positive predictive value of 80%, and negative predictive value of 75.5%). The overall accuracy was 77%. The OR for having MAG in patients with 8-OHdG below the cutoff was 0.08 (*p* = 0.0002) (Figure 2).

Eight-OHdG levels were significantly higher in patients in whom *H. pylori* infection was present that had been previously treated compared to those with “pure” autoimmune gastritis (median 14.51 no. adducts/10^5^ dG, CI 95% 12.6–23.6 vs. 6.5, CI 95% 2.7–13.55, *p* = 0.01 by Mann–Whitney U-test).

No significant difference was observed in the levels of 8-OHdG between the groups of patients with dysplasia and those without dysplasia.

### Cytokine tissue expressions

IL-10 was higher in MAG patients compared to AAG patients (median values (IQR) 114.02 (198.57) vs. 45.0 (71.22), respectively; p = 0.008) and in MAG patients in the “early” and “advanced” OLGA stages when compared with the “early” and “advanced” OLGA stages in AAG patients (*p* = 0.01).

No statistically significant differences were observed in the comparison of TNFα levels between AAG and MAG patients and between early vs. advanced MAG patients, *H. pylori*+ vs. *H. pylori*- AAG patients, and ECL + vs. ECL – AAG patients.

With respect to IL-10 in AAG patients, a significant difference (using the Mann–Whitney U-test) was observed between patients with a history of *H. pylori* infection (median values 43.81, 95% C.I. 39.35–255.71) and those without *H. pylori* infection (median values 51.51, 95% C.I. 24.51–153.73).

Similarly, no significant differences were observed in TNFα levels (mean values 77.14 +/– 38.73 S.D. vs. 77.61 +/– 38.73 S.D., Student's T-test).

### Correlations

In AAG patients, a rank correlation analysis using Spearman's coefficient for non-parametric distribution was performed to assess the relationships between dROM levels, AOPPs, NO levels and bio-humoral parameters, identifying a correlation in the distributions between the dROMs and the AOPPs with *p* = 0.02. No significant correlations were observed in MAG patients. Furthermore, PGI/PGII ratio was significantly correlated with dROM levels in AAG patients with *p* = 0.004.

## Discussion

The presence of enzymatic and non-enzymatic antioxidant agents, which are capable of counteracting the excesses of free radicals (redox balance) (12), is fundamental for maintaining homeostasis in living organisms. Indeed, multiple damages can result from an excess of free radicals or a defect in antioxidants, including protein, lipid, and nucleic acid damage (12). It has been hypothesized that the alteration of this redox balance is involved in the development and maintenance of numerous diseases, including autoimmune diseases (12). Furthermore, the damage resulting from oxidative stress triggers and maintains carcinogenic processes, as has been demonstrated in previous studies on gastric and esophageal cancer (20, 25, 26). Notably, oxidative damage can be assessed by measuring the 8-OHdG levels, a DNA adduct found to be high in gastric cancer. Cytokines are involved in the carcinogenetic and autoimmune processes, as they are responsible for the pro-/anti-inflammatory balance.

The presence of gastric atrophy is a well-known preneoplastic condition, even though the risk of progression toward cancer is different according to the type of gastritis (3, 4). The risk of adenocarcinoma development in AAG is lower compared to atrophic multifocal *H. pylori-related* gastritis. However, in AAG, there is an increased risk of developing type I carcinoids, resulting from the hyperplasia of ECL cells in response to the reduction or absence of gastric acid secretion (4). The role of oxidative stress, through the evaluation of AOPPs, oxidative genomic damage, and nitrosative damage, has already been evaluated in *H. pylori* atrophic gastritis and gastric cancer, highlighting an increase of these parameters compared to healthy controls (20). However, to date, no studies have ever assessed the role of oxidative stress and its pro-oncogenic role in AAG patients. Therefore, in this study, we compared damage due to oxidative stress in AAG patients and that in MAG patients.

The epidemiological and pathologic features of these patients agree with the literature, with a higher prevalence of female patients (27) and the absence of concomitant or previous dysplastic/neoplastic lesions in AAG (4, 28), in contrast to the findings of MAG patients. Both MAG and AAG patients present high levels of oxidative stress-related parameters, without significant differences between the two groups in dROM, NO, and AOPP levels. Considering the risk of cancer progression in MAG patients and of the development of neuroendocrine tumor in AAG patients, we sub-grouped patients in “early” OLGA stages (MAG 1–2 and AAG 0–1) and advanced OLGA stages (MAG 3–4 and AAG 2–4). Interestingly, “early” AAG demonstrated higher levels of dROMs when compared with “advanced” stages, and a difference was also detected in comparing “early” AAG stages with “early” MAG stages. Moreover, in AAG patients, high levels of dROMs and AOPPs were demonstrated in the absence or the early stages of ECL hyperplasia. These results lead us to hypothesize an early role of oxidative stress in the development of gastric damage in AAG. While in the advanced stages of damage, the target of oxidative processes (gastric mucosa) disappears due to the atrophic processes, in early gastritis, a residual mucosal glandular target is still present for oxidative stress action. Once the mucosa is completely atrophic, the production of free radicals appears to decline. This hypothesis was supported by the correlation between dROM levels and PGI/PGII as well as a trend toward a rank correlation between dROM levels and AOPP levels, with findings not confirmed in MAG patients, where damage elicits a different type of inflammation.

In fact, AOPP levels, which reflect an oxidative result in AAG, can induce neo-antigen formation (15). Consequently, the role of AOPPs in the “early” phases of AAG may be attributed either to the presence of residual mucosal tissue or to oxidative stress, maintaining the autoimmune reaction.

Finally, with respect to NO levels, no significant differences were observed between AAG and MAG patients, also when analyzed atrophic stages and H-ECL (in AAG). Moreover, high and toxic levels of NO were found in both MAG and AAG patients, compared to what was described in the literature.

It is probable that the most intriguing finding is the very low presence of 8-OHdG in AAG patients compared to what is documented in MAG patients. This adduct is mutagenic and indicates the extent of genomic oxidative damage. It is considered a marker of cancer risk, and therefore, it is not surprising that this adduct is higher in MAG patients compared to AAG patients, where the risk of progression to adenocarcinoma is lower.

Similarly, when patients with AAG were sub-grouped into those who had an *H. pylori* infection vs. those who had no previous or concomitant *H. pylori* infection, the former group showed significantly higher levels of 8-OHdG, providing additional evidence of the relevance of *H. pylori* in causing an accumulation of genomic damage. Furthermore, our ROC curve analysis demonstrated that, with a relatively low cut-off value of 8-OHdG levels (28.1 no. adducts/10^5^ dG), we could differentiate patients with AAG from patients with MAG, with an overall accuracy (77%) and a particularly high specificity 95%. The OR for MAG patients with 8-OHdG levels below the cut-off value was 8%.

A study demonstrated how the differences between AAG and MAG can be explained by the different types of inflammation in the two different gastritis, represented by extensive macrophage infiltration in MAG, which is almost absent in AAG patients. Macrophages, mostly present in *H. pylori*-induced gastritis, are the most effective oxidative stress inducers, inducing a genomic damage-dependent progression toward cancer in MAG patients, which is clearly more effective than that toward cancer in AAG patients (29). Macrophages' mucosal infiltration is the type of inflammation that triggers the progression to dysplasia and cancer, as in the Correa cascade (10), and, in its absence, progression to cancer is considered a very uncommon event. In our study, we demonstrated how oxidative stress, well represented in both gastritis, appears to have a specific genomic target, the 8-OHdG genomic adduct, in MAG patients. In AAG patients, the genomic damage is probably not relevant, thus explaining the low risk of developing cancer in them compared to that in MAG patients.

An additional component of the inflammatory scenario in chronic atrophic gastritis is represented by the inflammatory cytokines in the milieu.

Considering the role of cytokines in oxidative stress, in the modulation of the inflammatory response, and in autoimmune processes, we indeed measured the tissue level of IL-10, an anti-inflammatory cytokine, and TNFα, a pro-inflammatory cytokine, in AAG and MAG patients. A statistically significant difference was observed in the overall median values of IL-10, with higher levels in MAG than in AAG patients, and in “advanced” MAG compared to “advanced” AAG patients. An interpretation of the overexpression of IL-10 in MAG patients, compared to AAG patients, may be attributed to the extent of chronic inflammation present in MAG, which is secondary to *H. pylori* infection and associated with macrophage recruitment. It could be hypothesized that the gastric mucosa, under the inflammatory status induced by *H. pylori* infection, implements both defensive and anti-inflammatory systems (among which IL-10) to a larger extent than autoimmune inflammation. The absence, on the other hand, of statistically significant differences in the tissue production of TNFα leads to the hypothesis that, regardless of the stimulus and the mechanism of inflammation, there is a significant pro-inflammatory activity, whether macrophage- or lymphocyte-mediated. It must be, however, underlined that both IL-10 and TNFα may have both a pro- and an anti-tumor properties with respect to gastric carcinogenesis (30) and that, therefore, our findings should be cautiously considered.

### Study limitations

This study presents some intrinsic and extrinsic limitations. With respect to the intrinsic limitations, one limitation is represented by the retrospective nature of the study, even though patients were not “selected” for our study but consecutively enrolled, which reduces the impact of the retrospective nature.

The relatively small number of patients might be considered as a limitation for the generalization of the data obtained. However, even though numerous clinical studies have been recently published with a large number of patients with autoimmune gastritis, none can be identified in this field on the large number in the last 3 years with respect to basic science aspects. Furthermore, the lack of patients with end-stage disease, in terms of cancer progression, limits the final considerations, since our data are all referring to patients with precancerous changes. Finally, another limitation of this study is the lack of investigations on the activity of gastric anti-oxidant systems.

However, this study, being the first to evaluate the possible targets of oxidative stress and inflammation in AAG, paves the way for investigating a previously unknown pathway, starting from inflammation and leading to oxidative damage (protein or genomic), in AAG and MAG patients. Additional studies are needed to confirm our data, in particular, with the inclusion of a larger series of *H. pylori*-related MAG.

## Data Availability

The raw data supporting the conclusions of this article will be made available by the authors, without undue reservation.
